# Supplementary material for: Influence of Carbon Sources on the Phenolic Compound Production by Euglena gracilis Using an Untargeted Metabolomic Approach
Source: Biomolecules. 2022 Jun 7;12(6):795. doi: 10.3390/biom12060795 (PMC9221438; doi:10.3390/biom12060795)
Supplement: Supplementary file 1 [file biomolecules-12-00795-s001.zip › biomolecules-1736281-supplementary.pdf]

INFLUENCE OF CARBON SOURCES ON THE PHENOLIC COMPOUND PRODUCTION BY EUGLENA GRACILIS USING AN UNTARGETED METABOLOMIC APPROACH

Eve Bernard[1], Céline Guéguen[1,*]

[1] Université de Sherbrooke, Département de chimie, Sherbrooke, QC, CANADA

*corresponding author: celine.gueguen@usherbrooke.ca

Supplemental information
Tables S1-S2
Figures S1-S7

Table S1. Retention time and linearity of the phenolic standards

Standard	Retention time (± 0.1min)	R^2
Gallic acid	2.7	1.0000
Cathechin	4.8	0.9999
Chlorogenic acid	5.4	0.9999
Caffeic acid	5.9	0.9995
P-Coumaric acid	6.4	0.9999
Ferrulic acid	8.1	0.9998
Naringenin	10.8	0.9999
Quercetin	11.0	0.9999
Kaempferol	12.3	1.0000

Table S2. Weighted average of elemental ratios and indices of the five LC-MS fractions F1-F5.

Carbon source	Fraction	O/C	H/C	AImod	DBE	NOSC
Glutamic acid	1	0.429 ± 0.016	1.057 ± 0.088	0.443 ± 0.060	1.333 ± 0.350	0.058 ± 0.097
	2	0.488 ± 0.043	0.887 ± 0.091	0.509 ± 0.069	2.000 ± 0.667	0.460 ± 0.040
	3	0.481 ± 0.046	1.333 ± 0.000	-0.412 ± 0.416	-0.500 ± 0.354	0.500 ± 0.589
	4	0.574 ± 0.020	1.092 ± 0.032	0.340 ± 0.014	0.016 ± 0.459	0.193 ± 0.097
	5	0.505 ± 0.006	1.165 ± 0.010	0.305 ± 0.008	-0.828 ± 0.016	0.401 ± 0.027
Glucose	1	0.919 ± 0.224	1.874 ± 0.376	0.302 ± 0.143	-1.164 ± 0.389	0.671 ± 0.185
	2	0.621 ± 0.056	1.013 ± 0.018	0.443 ± 0.023	0.091 ± 0.392	0.428 ± 0.044
	3	0.581 ± 0.019	1.353 ± 0.020	0.019 ± 0.089	-1.959 ± 0.347	0.392 ± 0.130
	4	0.628 ± 0.032	1.098 ± 0.101	0.167 ± 0.137	-1.527 ± 0.458	0.472 ± 0.044
	5	0.589 ± 0.026	1.102 ± 0.056	0.188 ± 0.068	-1.152 ± 0.404	0.411 ± 0.038
Malic acid	1	0.509 ± 0.009	0.884 ± 0.043	0.534 ± 0.036	0.866 ± 0.236	0.300 ± 0.041
	2	0.530 ± 0.010	0.993 ± 0.088	0.456 ± 0.062	0.419 ± 0.437	0.091 ± 0.102
	3	0.557 ± 0.012	0.978 ± 0.081	0.467 ± 0.061	0.020 ± 0.442	0.193 ± 0.101
	4	0.521 ± 0.010	0.927 ± 0.015	0.508 ± 0.009	1.085 ± 0.080	0.257 ± 0.029
	5	0.490 ± 0.019	1.058 ± 0.033	0.397 ± 0.023	0.370 ± 0.117	0.100 ± 0.050
GSH	1	0.501 ± 0.056	0.510 ± 0.170	0.365 ± 0.200	0.800 ± 0.395	0.191 ± 0.155
	2	0.439 ± 0.072	0.878 ± 0.116	0.342 ± 0.059	0.118 ± 0.360	0.209 ± 0.109
	3	0.424 ± 0.090	0.657 ± 0.133	0.411 ± 0.092	0.833 ± 0.130	0.279 ± 0.070
	4	0.429 ± 0.058	0.702 ± 0.099	0.383 ± 0.058	0.371 ± 0.162	0.246 ± 0.073
	5	0.410 ± 0.048	0.808 ± 0.116	0.279 ± 0.066	0.000 ± 0.358	0.238 ± 0.048

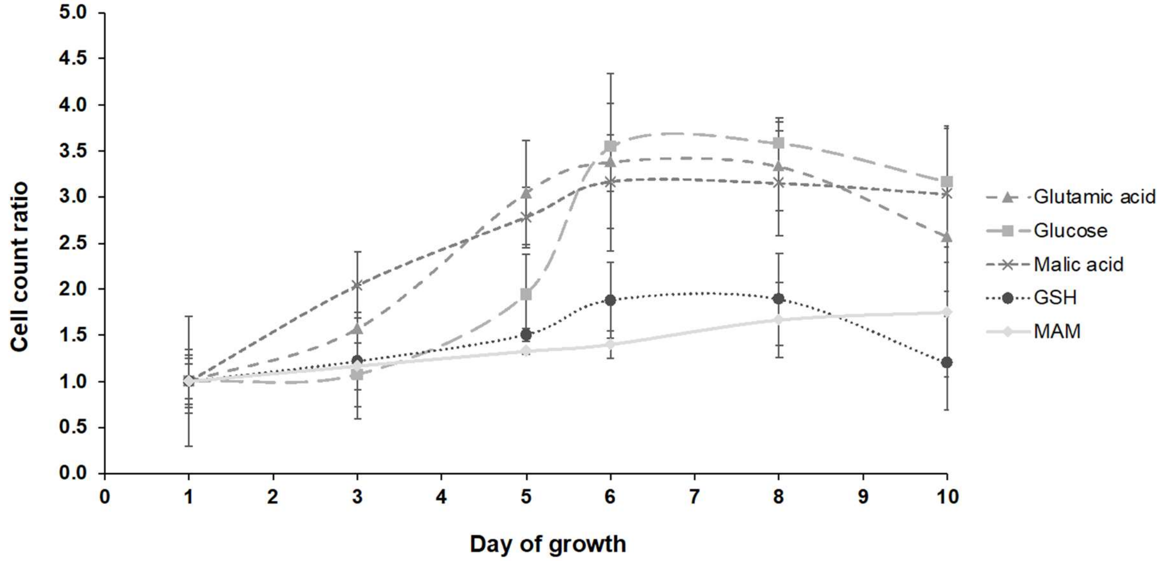

Figure S1. Growth curves in inorganic medium supplemented with carbon sources.

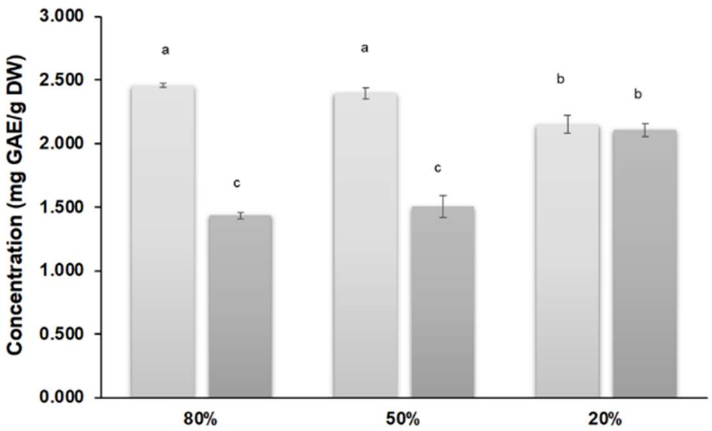

Figure S2. Concentrations of gallic acid equivalent (GAE) found in methanol and ethanol extracts (light and dark gray, respectively). Different superscript letters indicate significant differences ($p<0.05$) determined by Wilcoxon test.

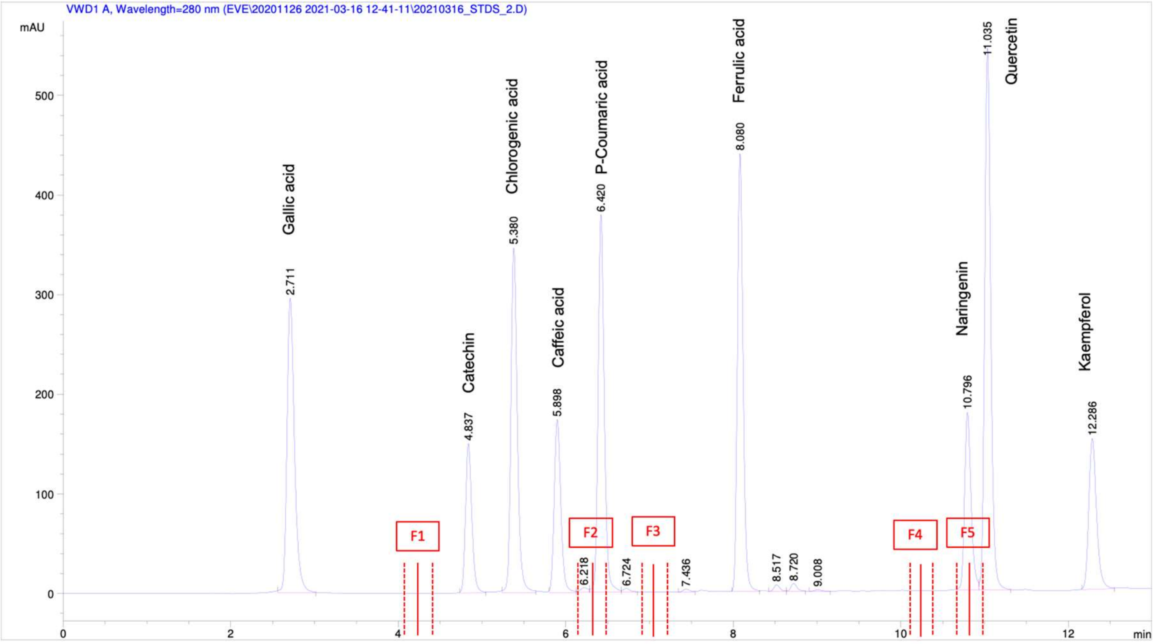

Figure S3. Chromatogram of the phenolic standards. F1-F5 indicate the collected fractions in MS analyses

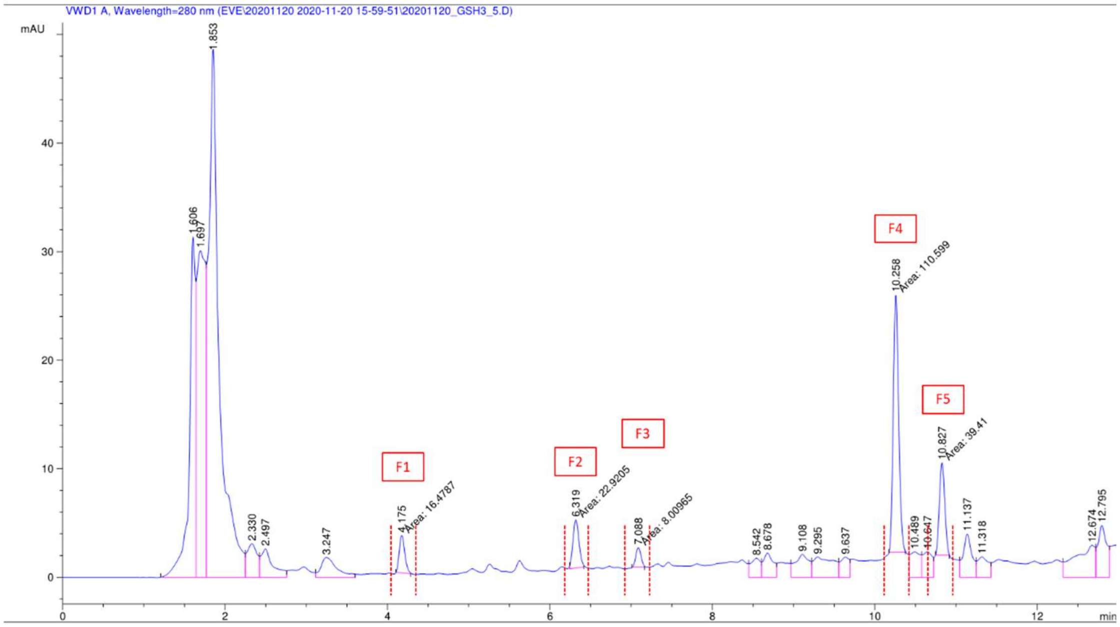

Figure S4. Chromatogram of a cellular extract on day 3 of growth in de MAM medium supplemented with GSH. F1-F5 represent the collected fractions used in MS analyses

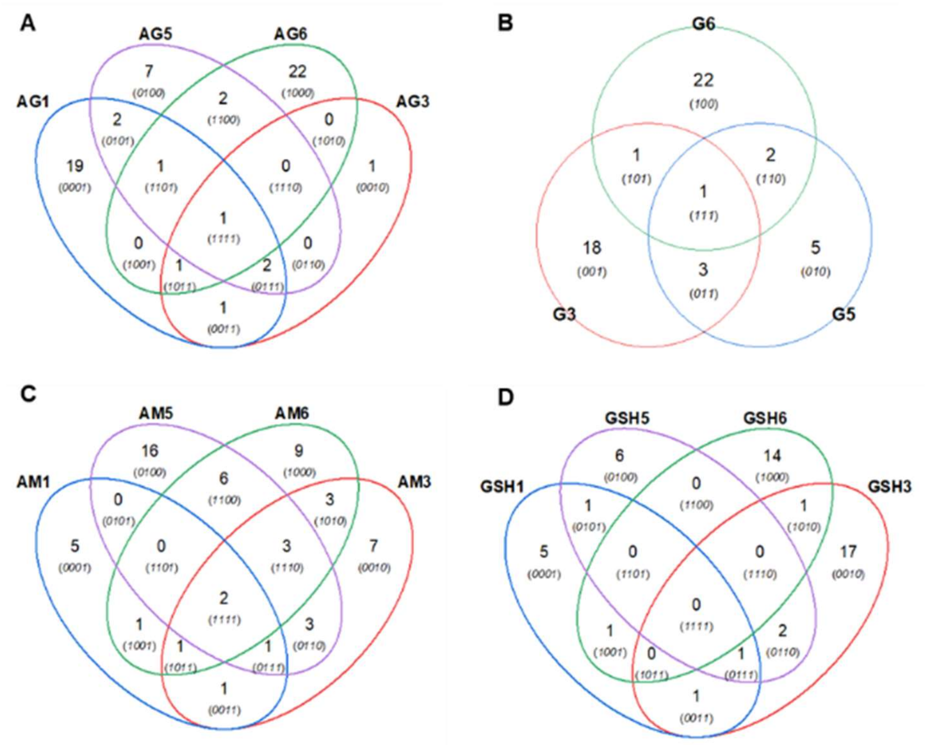

Figure S5. Venn diagram of the molecular formulas attributed for each day of the lag phase in the **(A)** glutamic acid, **(B)** malic acid, **(C)** glucose, and **(D)** GSH amendment. The digit 1 and 0 indicating the groups represented, each group having a specific digit 1 position.

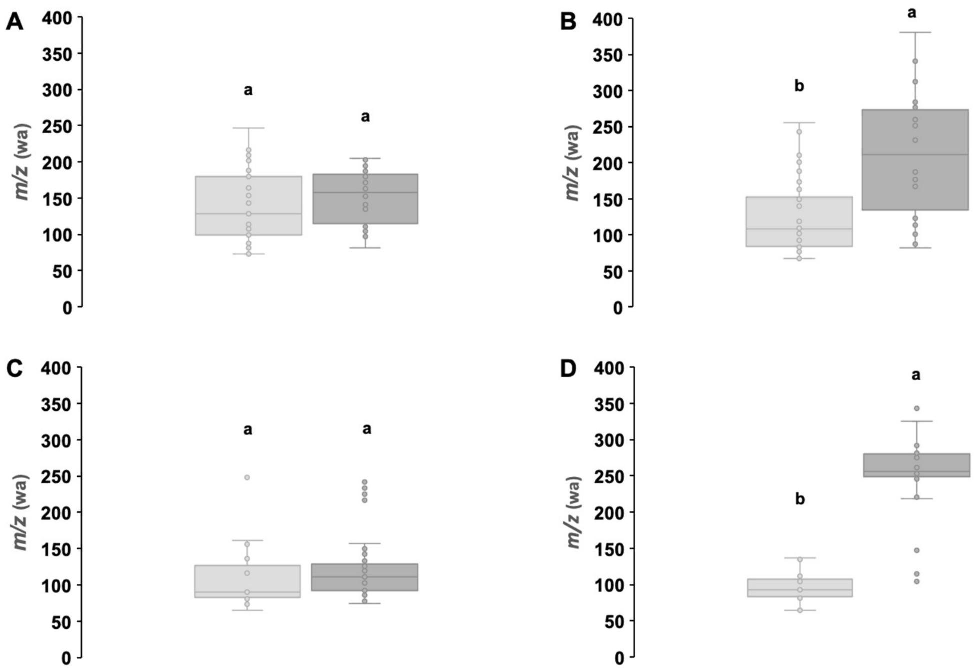

Figure S6. Box plot of all the weighted average (wa) *m/z* found at the start and at the end of the log phase in (A) glutamic acid, (B) glucose, (C) malic acid, and (D) GSH amendment. Different superscript letters indicate significant differences ($p<0.05$) determined by Wilcoxon test.

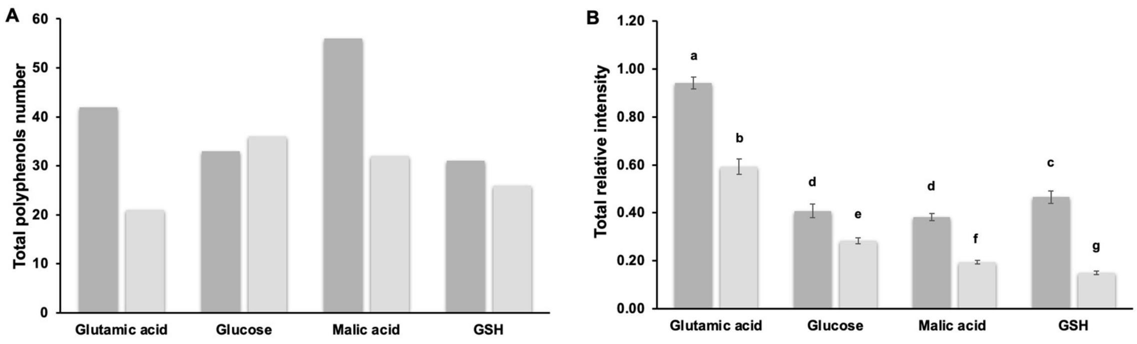

Figure S7. (A) Number of polyphenols and (B) their relative intensities associated with the minimum growth rate and the maximum growth rate. Different superscript letters indicate significant differences ($p<0.05$) determined by Wilcoxon test.
